# Edoxaban use in the context of dental procedures: analysis from the EMIT-AF/VTE database

**DOI:** 10.1038/s41405-023-00164-6

**Published:** 2023-08-14

**Authors:** Cathy Chen, Manish Saxena, Christian von Heymann, Thomas Vanassche, James Jin, Robert Lersch, Sabine Köhler, Amparo Santamaria, Martin Unverdorben, Paolo Colonna

**Affiliations:** 1 Daiichi Sankyo, Inc., Basking Ridge, NJ USA; 2 Barts NIHR Cardiovascular Biomedical Research Centre, London, UK; 3 Department of Anaesthesia & Intensive Care Medicine, Emergency Medicine, and Pain Therapy, Vivantes Klinikum im Friedrichshain, Berlin, Germany; 4 Department of Cardiovascular Sciences, University Hospitals (UZ) Leuven, Leuven, Belgium; 5 Serenis GmbH, Munich, Germany; 6 Hematology Department, University Hospital Vinalopó y Torrevieja, Alicante, Spain; 7 Department of Cardiology, Polyclinic of Bari – Hospital, Bari, Italy

**Keywords:** Dental implants, Warfarin in dentistry

## Abstract

**Introduction:**

Literature reviews support continuing anticoagulation during dental procedures. However, studies often present grouped anticoagulation data, and information on individual anticoagulant management would be helpful to dentists. The Edoxaban Management in Diagnostic and Therapeutic Procedures (EMIT-AF/VTE) programme (NCT02950168; NCT02951039) demonstrated low periprocedural bleeding and thrombotic event rates in patients with atrial fibrillation receiving edoxaban.

**Aims:**

To report periprocedural edoxaban interruption and clinical events in patients from EMIT-AF/VTE who underwent dental procedures.

**Methods:**

Dental procedures were categorised by type (cleaning/noncleaning). Edoxaban interruption, bleeding events, and thrombotic events were observed 5 days preprocedure through 29 days postprocedure.

**Results:**

Overall, 196 patients underwent 350 cleaning and/or noncleaning procedures; most patients (171/196 [87.2%]) underwent noncleaning procedures (282/350 [80.6%]), whereas 48/196 (24.5%) underwent 68/350 (19.4%) cleaning procedures. Edoxaban was uninterrupted for most cleanings (53/68 [77.9%]). Preprocedural interruption was common for single and multiple tooth extractions (single, 67/100 [67.0%]; multiple, 16/30 [53.3%]). The only major bleeding occurred after an unrelated cleaning. Minor bleeding occurred in 1/68 (1.5%) cleaning and 4/282 (1.4%) noncleaning procedures. There were no thrombotic events.

**Conclusions:**

For most cleanings, edoxaban was not interrupted, whereas preprocedural interruption was more common for tooth extractions. Overall, bleeding rates were low, and no thrombotic events occurred.

## Introduction

Patients who are treated with anticoagulants—commonly prescribed for stroke prevention in patients with atrial fibrillation (AF) and for the secondary prevention of venous thromboembolism (VTE)—may have an increased risk of bleeding following dental cleanings or other common dental procedures, as these therapies delay the formation of clots and increase the risk of procedure-related bleeding [1, 2]. For decades, vitamin K antagonists (VKAs) were the only available class of oral anticoagulants [2]. Because of their slow onset and offset of effect, interrupting and restarting VKA therapy is challenging and increases the risk of bleeding and thrombotic events [3, 4]. Early studies of periprocedural VKA use focused on the bleeding and thrombotic event risk without interruption and demonstrated that if patients are on a well-managed VKA regimen (international normalised ratio <3.5) and local haemostatic measures are adequate, continuous anticoagulation with VKAs during dental procedures is at least as safe as interruption [5].

Based on prior studies with VKAs and clinician experience with performing dental procedures without interrupting anticoagulants, continuous anticoagulation with non–vitamin K oral anticoagulants (NOACs; also known as direct oral anticoagulants) is also recommended, which is supported by head-to-head studies demonstrating similar or reduced bleeding rates with NOACs vs VKAs for dental procedures [5–7]. However, because of their short half-life and fast onset and offset, brief periprocedural interruption of NOAC treatment is easier than interruption of VKA therapy [3]. Furthermore, NOAC-specific treatment guidelines recommend that physicians briefly interrupt NOAC treatment before, on the day of, or after a variety of procedures, including complex dental procedures [8]. However, there is a paucity of data that report bleeding risk for NOACs across a variety of dental procedures, and there is insufficient evidence available to guide decision making for individual NOACs. This is a concern as NOACs are now the standard of care for stroke prevention in patients with AF, and with rising numbers of NOAC prescriptions, dental professionals will treat an increasing number of patients receiving NOACs [9, 10].

While the literature generally supports the continued use of NOACs during dental procedures, information specific to patient bleeding tendencies associated with individual NOACs, including edoxaban, would be helpful to practitioners. To address this knowledge gap, we present a subgroup analysis of patients from the Edoxaban Management in Diagnostic and Therapeutic Procedures (EMIT-AF/VTE) programme (NCT02950168; NCT02951039) who underwent dental procedures.

## Methods

The protocol design and overall results of the EMIT-AF/VTE programme were published previously [11, 12]. EMIT-AF/VTE was a multicentre, prospective, observational programme conducted in Europe and Asia in accordance with the Declaration of Helsinki and with the approval of local institutional review boards [11]. Written informed consent was obtained prior to enrolment [12]. Patients with AF or VTE underwent diagnostic or therapeutic procedures while prescribed and receiving edoxaban in routine clinical practice for stroke prevention [11]. Edoxaban therapy was managed at the discretion of the investigator [12]. Eligible patients were those ≥18 years of age with AF or VTE; patients were treated with edoxaban according to local labels [11]. Patients in this subanalysis were grouped by whether they underwent dental cleaning and/or noncleaning procedures. Those with both cleaning and noncleaning procedures were counted in both groups in the summary of baseline demographics and clinical characteristics. Edoxaban interruption and clinical outcomes were summarised per procedure.

The observation period for cleaning and noncleaning procedures (tooth extraction [single or multiple], dental interventions [root canal/resection, dental implant placements, crown replacements], and other noncleaning procedures) in this subanalysis began 5 days preprocedure and ended 29 days postprocedure. Periprocedural edoxaban interruption was categorised by its occurrence before (preprocedure only), after (postprocedure only), or both before and after the procedure (pre- and postprocedure). The duration of edoxaban interruption was calculated only for procedures with any periprocedural interruption (i.e., procedures without interruption were excluded). The primary outcome was incidence of major bleeding as defined by the International Society of Thrombosis and Haemostasis (ISTH) [12, 13]. Secondary outcomes included clinically relevant nonmajor (CRNM) bleeding, defined as overt bleeding requiring medical attention but not fulfilling criteria for major bleeding, and minor bleeding [11, 12]. Key demographic data, concomitant medications, edoxaban treatment details, and clinical findings were collected per protocol [11]. Results were reported as summary statistics [11, 12].

## Results

A total of 196 patients had cleaning (*n* = 48 [24.5%]) and/or noncleaning dental procedures (*n* = 171 [87.2%]; Table 1). Demographics were well balanced between groups, with a slightly higher proportion of males and numerically lower mean age for patients who received dental cleanings vs those who received noncleaning procedures (Table 1). However, those receiving more invasive, noncleaning procedures vs patients with cleanings had higher rates of diabetes mellitus (24.0% vs 12.5%), renal disease (13.5% vs 4.2%), congestive heart failure (11.7% vs 2.1%), and creatinine clearance ≤50 mL/min (18.7% vs 4.2%) and were more likely to be on a reduced edoxaban dose regimen of 30 mg/day (26.3% vs 16.7%).

**Table 1:** Baseline demographics and clinical characteristics in patients who underwent dental procedures in the EMIT-AF/VTE programme.

	Cleanings (*n* = 48)	Noncleaning procedures^a^ (*n* = 171)
Age, mean ± SD, years	71.5 ± 7.5	72.3 ± 10.4
<65 years	5 (10.4)	33 (19.3)
65–74 years	28 (58.3)	63 (36.8)
≥75 years	15 (31.3)	75 (43.9)
Sex, male	35 (72.9)	104 (60.8)
Weight, mean ± SD, kg	79.2 ± 12.1	78.1 ± 17.3
BMI, mean ± SD, kg/m^2^	27.5 ± 3.7	27.5 ± 5.0
CrCl, mean ± SD, mL/min	73.0 ± 17.3	72.5 ± 33.6
CrCl ≤50 mL/min	2 (4.2)	32 (18.7)
Indication for edoxaban		
Atrial fibrillation	44 (91.7)	156 (91.2)
Venous thromboembolism	3 (6.3)	18 (10.5)
Medical history		
Hypertension	43 (89.6)	139 (81.3)
Dyslipidaemia	21 (43.8)	75 (43.9)
Diabetes mellitus	6 (12.5)	41 (24.0)
Coronary heart disease	6 (12.5)	23 (13.5)
Valvular heart disease	8 (16.7)	19 (11.1)
Renal disease	2 (4.2)	23 (13.5)
Congestive heart failure	1 (2.1)	20 (11.7)
Heparin (including LMWH)	1 (2.1)	4 (2.3)
Antiplatelet agents	7 (14.6)	22 (12.9)
HAS-BLED score, mean ± SD	1.7 ± 1.1	1.7 ± 1.0
CHA_2_DS_2_-VASc score, mean ± SD	2.9 ± 1.1	3.4 ± 1.6
Edoxaban regimen		
30 mg/day	8 (16.7)	45 (26.3)
60 mg/day	40 (83.3)	126 (73.7)

Data presented as *n* (%) unless otherwise indicated. Patients with both cleaning and noncleaning procedures were counted in both groups.

*BMI* body mass index, *CHA*_*2*_*DS*_*2*_*-VASc* congestive heart failure, hypertension, age ≥75 years, diabetes mellitus, stroke or transient ischaemic attack, vascular disease, age 65–74 years, sex category, *CrCl* creatinine clearance, *HAS-BLED* hypertension, abnormal renal/liver function, stroke, bleeding history or predisposition, labile international normalised ratio, elderly, drugs/alcohol concomitantly, *LMWH* low-molecular-weight heparin, *SD* standard deviation.

^a^Includes tooth extraction (single or multiple), dental implant placement/tooth implantation, crown placement, root canal, and others (e.g., mandibular surgery).

In all, patients receiving edoxaban underwent 350 dental procedures (Table 2). There were 68 cleaning procedures and 282 noncleaning procedures (Table 2). The most common noncleaning procedures included single tooth extractions (*n* = 100 [35.5%]), dental implant placements (*n* = 45 [16.0%]), ‘other’ (*n* = 37 [13.1%]), crown placements (*n* = 33 [11.7%]), multiple tooth extractions (*n* = 30 [10.6%]), root canals (*n* = 25 [8.9%]), and tooth implantations (*n* = 13 [4.6%]). Less-common procedures (*n* ≤3 [≤1.1%]) included transmandibular/transmaxillar root resection, orthodontic procedures, mandibular surgery, caries removal and resin filling; crown impression, lengthening, preparation, and deletion; and stitches and dressing.

**Table 2:** Bleeding events in patients who underwent dental procedures in the EMIT-AF/VTE programme.

	Cleanings (*n* = 48)	Noncleaning procedures^a^ (*n* = 171)
Number of procedures	68	282
All bleeding events, *n* (%)	2 (2.9)	4 (1.4)
Major bleeding^b^	1 (1.5)	0
Minor bleeding	1 (1.5)	4 (1.4)
CRNM bleeding	0	0

Patients with both cleaning and noncleaning procedures were counted in both groups. Percentages were calculated per number of procedures.

*CRNM* clinically relevant nonmajor bleeding.

^a^Includes tooth extraction (single or multiple), dental implant placement/tooth implantation, crown placement, root canal, and others (e.g., mandibular surgery).

^b^The major bleeding event was intraocular after an ophthalmology procedure and not related to the dental procedure. Per the EMIT-AF/VTE observational plan, this event is included in the subanalysis.

Of the 68 cleanings, edoxaban was not interrupted in most cases (*n* = 53 [77.9%]). When edoxaban therapy was interrupted, it was typically either preprocedure only or postprocedure only (*n* = 6 [8.8%] for both); edoxaban was stopped both before and after 3 (4.4%) cleanings. The mean ± standard deviation (SD) interruption time for cleanings was 4.5 ± 8.6 days (median, 2 days).

Among all noncleaning procedures (*n* = 282), edoxaban was not interrupted in approximately half (*n* = 139 [49.3%]). When investigators decided to interrupt edoxaban for a noncleaning procedure, it was most often only preprocedure (*n* = 110 [39.0%]). Edoxaban was less frequently stopped both before and after the procedure (*n* = 25 [8.9%]) or only postprocedure (*n* = 8 [2.8%]). The mean ± SD duration of edoxaban interruption for noncleaning procedures was 2.6 ± 3.4 days (median, 1 day).

**Fig. 1:**
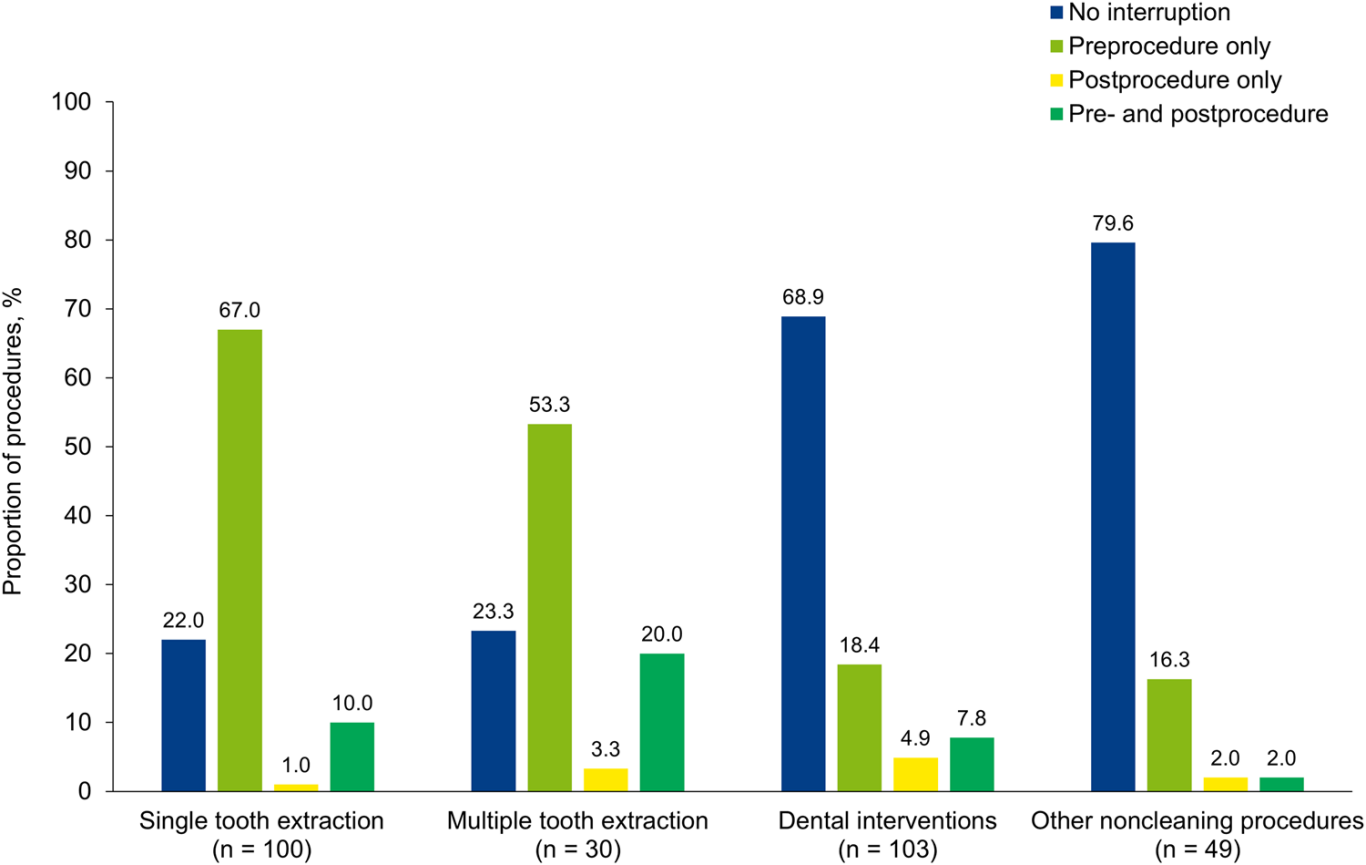
Edoxaban interruption for noncleaning dental procedures in the EMIT-AF/VTE programme. The proportion of noncleaning procedures with no edoxaban interruption, preprocedure, postprocedure, or pre- and postprocedure interruption are presented.

When noncleaning procedures were subdivided into categories of single tooth extractions, multiple tooth extractions, dental surgery, and other noncleaning procedures, edoxaban management strategies diverged (Fig. 1). Among dental extractions (single and multiple), edoxaban therapy was usually interrupted, most often before the procedure (Fig. 1). The mean ± SD duration of edoxaban interruption was similar between single tooth extractions (2.4 ± 3.6 days; median, 1 day) and multiple tooth extractions (2.6 ± 1.6 days; median, 2 days). In contrast to dental extractions, most dental interventions (*n* = 71/103 [68.9%]) and other noncleaning procedures (*n* = 39/49 [79.6%]) proceeded without edoxaban interruption (Fig. 1). When edoxaban was interrupted for a dental intervention, the mean ± SD duration was 2.8 ± 4.2 days (median, 2 days).

There were 78 total dental implant–related procedures, comprising 45 dental implant placements and 33 crown placements. Edoxaban was interrupted in almost half of the dental implant placements (*n* = 20/45 [44.4%]), but only in a minority of crown placements (*n* = 5/33 [15.2%]). If edoxaban was interrupted for a dental implant placement, it was most often before the procedure (*n* = 11/45 [24.4%]). Edoxaban was less frequently stopped both pre- and postprocedure (*n* = 8/45 [17.8%]), with 1 interruption occurring only after the procedure. Among crown placements (*n* = 33), preprocedural interruption occurred in 3 (9.1%) crown placements, none had both pre- and postprocedural edoxaban interruption, and 2 (6.1%) were interrupted only postprocedure. The mean ± SD duration of edoxaban interruption for dental implant placements was 3.2 ± 5.0 days (median, 2 days), whereas crown placements had a shorter interruption time of 1.8 ± 1.3 days (median, 1 day).

Bleeding was rare for cleaning procedures, with 1 minor bleeding and 1 major bleeding event (Table 2). The major bleeding event was an intraocular bleed following an ophthalmology procedure (tarsal strip/punctoplasty) and was not related to the dental procedure. Per the observational plan of the EMIT-AF/VTE programme, we include this major bleeding event in the subanalysis since it occurred 28 days after a cleaning (i.e., within the 29-day postprocedural observation window). The minor bleeding event was within 7 days of the cleaning. The rate of any bleeding was 2.9 per 100 cleanings and 1.4 per 100 noncleaning procedures (Table 2). All bleeding events for noncleaning procedures (*n* = 4) were minor: 2 were associated with single tooth extractions, 1 with tooth implantation, and 1 reported as ‘other’. Two events were within 7 days of the procedure (single tooth extraction and tooth implantation), 2 each occurred with and without edoxaban interruption (with interruption: single tooth extraction and tooth implantation; without interruption: single tooth extraction and ‘other’), and no CRNM bleeding was observed for any dental procedure (Table 2). Only 1 event, gingiva irritated bleeding provoked on the same day as single tooth extraction, was related to the dental procedure, and the individual had preprocedural edoxaban interruption. No thrombotic events were recorded for any cleaning or noncleaning dental procedure.

## Discussion

This subanalysis from the EMIT-AF/VTE programme analysed dental procedures performed on patients receiving edoxaban in routine clinical practice. Of interest was whether edoxaban therapy was interrupted for dental procedures, the timing of interruption if it occurred, and the resulting clinical outcomes. The edoxaban management strategy for dental cleanings was generally to continue treatment throughout the procedure, whereas more invasive procedures were more frequently associated with edoxaban interruption. Most interruptions were short and consisted of skipping 1 or 2 doses before the procedure (i.e., preprocedural interruption). Overall, the safety results from this subanalysis demonstrate that dental procedures had a low risk of bleeding and that most bleeding was minor. The sole major bleeding event was recorded in the 29-day postprocedural observation window for a dental cleaning but was definitely not related, as it occurred during an ocular procedure performed 28 days after the cleaning.

The bleeding event rates that occurred within 7 days of dental procedures in this subanalysis were consistent with those observed in other observational studies using the typical 7-day postprocedure follow-up window [14–16]. However, clinical events in EMIT-AF/VTE were recorded up to 29 days postprocedure, which allowed for greater sensitivity to detect ischaemic/thrombotic events. Importantly, no thrombotic events were observed in this subanalysis, suggesting that the edoxaban management strategies adopted by dental professionals did not increase the risk of stroke for dental procedures, regardless of how or whether edoxaban was interrupted.

The DENTST study investigated the outcomes of 86 patients receiving NOACs (all NOACs except edoxaban; 145 total teeth extracted) and 21 patients receiving warfarin (50 total teeth extracted) [14]. In both treatment arms, no major bleeding events were reported during the 7-day follow-up period [14]. The rate of minor plus CRNM bleeding in the NOAC treatment group (36%) was similar to that observed in the warfarin control cohort (43%) [14]. The authors concluded that there was no need to adjust NOAC dosing prior to dental extraction [14].

Another prospective observational study with a postprocedural observation window of 7 days assessed outcomes of dental extractions for 119 patients receiving NOACs (*n* = 128 extractions), including edoxaban, and 248 patients receiving warfarin (*n* = 262 extractions) [15]. Postprocedural bleeding rates were low, with a trend towards fewer events with NOACs (4/128 [3.1%]) vs warfarin (23/262 [8.8%]); thus, the authors concluded it was not necessary to interrupt NOAC therapy for tooth extractions [15]. A retrospective cohort study with an observation window 30 min preprocedure to 7 days postprocedure reported similar results, demonstrating no differences in bleeding risk between NOACs and VKAs, despite a different definition of bleeding compared with other studies and the current subanalysis of the EMIT-AF/VTE programme [16]. Patients (*N* = 541) underwent 634 tooth extraction procedures (1196 teeth extracted) [16]. Seventy-two extractions (*n* = 41 procedures) involved NOACs (all 4 NOACs were represented in the study), *n* = 100 (50 procedures) involved VKAs, and *n* = 1024 extractions (543 procedures) involved no anticoagulants [16]. The incidences of postextraction bleeding (incidence per tooth) were 10.4% for NOACs, 12.0% for VKAs, and 0.9% for no anticoagulants [16]. Bleeding risk was not significantly different between NOAC and VKA groups (*P* = 0.49) [16].

Bajkin et al. reported postoperative bleeding in 3.3–5.7% of patients on any anticoagulant, which is consistent with the rate of bleeding observed with edoxaban use during dental procedures in this subanalysis of EMIT-AF/VTE [6]. Few examples of bleeding were observed during implant procedures, and these cases were well controlled with haemostatic measures [6]. The authors concluded that the accumulating data on management strategies strongly favour continuing anticoagulation before, during, and immediately after invasive dental procedures [6]. Collectively, the evidence in this review and other studies suggests similar or reduced bleeding risk during dental procedures with NOACs vs VKAs [6, 7, 14–16]. Notably, these studies generally did not describe management strategies aside from continuous anticoagulation and were not able to distinguish whether interruption patterns varied by anticoagulant [6, 7, 14–16]. While most studies recommended continuing anticoagulation through dental procedures, clinical practice regarding edoxaban management varied by procedure type, and some form of interruption was common, underscoring the need for studies reporting interruption patterns and outcomes of dental procedures for individual NOACs [6, 7, 14–16].

The European Heart Rhythm Association (EHRA) 2021 practical guide considers minor-risk dental procedures, such as dental extractions (1–3 teeth), paradental surgery, implant positioning, and subgingival scaling/cleaning, at minor risk of bleeding and does not recommend anticoagulant interruption [8]. Other more complex dental procedures categorised as low risk require an assessment of risk based on individual patient clinical characteristics and may or may not warrant interruption [8]. In the present study, edoxaban was typically interrupted during single and multiple tooth extractions. This is consistent with a 2019 survey of dental practitioners, which found that 62% would interrupt NOAC therapy for single tooth extractions and 94% would continue VKA therapy [17].

The EHRA practical guide does not distinguish between dental implants and crown placements, although it would be expected that implants would be associated with a longer edoxaban interruption compared with less-invasive crown placements [8]. The granularity of the EMIT-AF/VTE data allowed for the distinction of these two procedures, and no bleeding events were recorded for any dental implants or crown placements, regardless of whether or not edoxaban was interrupted. Furthermore, the mean and median duration of edoxaban interruption was longer for dental implants than for crown placements, suggesting that within the broad EHRA recommendations for minor-risk procedures, there is variation in the edoxaban management strategies adopted by dental professionals in real-world practice for specific dental procedures.

The strengths of the current subanalysis of the EMIT-AF/VTE programme include its large number of patients receiving edoxaban, the use of the ISTH bleeding definition, and the 29-day postprocedural observation window. The study was limited by the fact that it was a retrospective analysis of a prospective registry; thus, periprocedural edoxaban management was not controlled by a study protocol, and no information regarding bleeding management was available. The observed low risk of bleeding is consistent with the EHRA designation of dental procedures as minor or low-risk interventions but complicates the identification of rare, high-risk patients that may become apparent in larger studies [8]. In all, this study addresses a gap in the published data, which generally group all NOACs, by providing edoxaban-specific results for anticoagulant interruption surrounding dental procedures in routine clinical practice.

## Conclusion

These results suggest that real-world practice for administering individual NOACs may vary from current guidelines, which do not recommend anticoagulant interruption for procedures with minor or low EHRA bleeding risk, and warrant further investigation. Overall, this subanalysis demonstrates that current edoxaban management strategies in real-world practice result in minimal bleeding across a variety of low-risk dental procedures.

## Data Availability

The data underlying this article cannot be shared publicly, as the Global EMIT-AF/VTE study is currently ongoing.
